# Pain and pain management in children and adolescents receiving hospital care: a cross-sectional study from Sweden

**DOI:** 10.1186/s12887-022-03319-w

**Published:** 2022-05-05

**Authors:** Viveka Andersson, Stefan Bergman, Ingela Henoch, Hanna Simonsson, Karin Ahlberg

**Affiliations:** 1 Sahlgrenska Academy, Institute of Health and Care Sciences, University of Gothenburg, Gothenburg, Sweden; 2 Department of Medicine, Halland Hospital Varberg, Träslövsvägen 68, 432 37, 432 81 Varberg, SE Sweden; 3 Primary Health Care Unit, Department of Public Health and Community Medicine, Institute of Medicine, Sahlgrenska Academy, University of Gothenburg, Gothenburg, Sweden; 4 Spenshult Research and Development Centre, Halmstad, Sweden; 5 Angered Local Hospital, Gothenburg, Sweden; 6 Department of Surgery, Halland Hospital Halmstad, Halmstad, Sweden

**Keywords:** Pain prevalence, Children, Pain management, Hospitalized, Pain assessment

## Abstract

**Background:**

Pain is a common symptom in children receiving hospital care. Adequate pain management in paediatric patients is of the utmost importance. Few studies have investigated children’s own experiences of pain during hospitalization.

**Aim:**

To describe the prevalence of pain, self-reported pain intensity at rest and during movement, pain management and compliance with pain treatment guidelines in children and adolescents receiving hospital care. Furthermore, to examine self-reported statements about pain relief and how often staff asked about pain.

**Methods:**

A quantitative, cross-sectional study with descriptive statistics as the data analysis method was conducted at a county hospital in western Sweden. Sixty-nine children/adolescents aged 6–18 years who had experienced pain during their hospital stay were included. A structured, verbally administered questionnaire was used to obtain pain reports. The participants were also asked what they considered alleviated pain and how often they told staff about pain. Patient demographics, prescribed analgesics and documentation of pain rating were obtained from medical records.

**Results:**

Fifty children/adolescents (72%) experienced moderate to severe pain in the previous 24 hours. At the time of the interview 36% reported moderate to severe pain at rest and 58% during movement. Seven participants (10%) reported severe pain both at rest and during movement. About one-third were on a regular multimodal analgesic regimen and 28% had used a validated pain rating scale. Thirty children/adolescents (43%) reported that they had experienced procedural pain in addition to their underlying pain condition. Most of the children/adolescents (74%) reported that analgesics provided pain relief. Forty (58%) stated that various non-pharmacological methods were helpful.

**Conclusions:**

Despite evidence-based guidelines, half of the children/adolescents experienced moderate to severe pain, highlighting the need for improvement. Pain levels should be assessed both at rest and during movement. Response to treatment should be evaluated to prevent undertreatment of pain. Compliance with guidelines and professional communication are of the utmost importance for pain management in children/adolescents. Non-pharmacological methods are a valuable part of a pain management strategy. This study shows that it is important to evaluate and improve pain care also outside specialised tertiary clinics.

## Background

Pain is a common symptom in children receiving hospital care [1]. Optimal pain treatment in children and adolescents is of the utmost importance, as inadequately managed acute pain can lead to chronic pain [2, 3] or posttraumatic stress symptoms [4]. Chronic pain has a negative impact on quality of life, which may have social and emotional consequences for children and their family members [5]. Pain is a multidimensional phenomenon with sensory, physiological, cognitive, affective and behavioural components [6]. Knowledge about pain in children has increased in recent decades. In the 1970s and 1980s, research began to explore subjective experiences of paediatric pain and children’s abilities to report their pain experiences [6]. Despite increased knowledge and guidelines [7, 8], investigations from the past 10 years show that moderate to severe pain is still common in hospitalized children and that analgesic regimens are not optimal [9–15]. Children and adolescents have the right to appropriate pain management treatment [7].

Non-pharmacological methods, such as psychological support and information, distraction, relaxation, massage and heat/cold therapy, are treatments used in children with acute and postoperative pain as well as with persistent disease-related and chronic pain [1, 7, 8, 16, 17]. A multimodal analgesic regimen consisting of two or more medications is recommended for children with persistent disease-related pain [17], as well as for those with acute and postoperative pain [1, 8, 16]. A variety of analgesic medications and techniques that target different mechanisms in the peripheral and/or central nervous system can provide more effective pain relief compared with single-modality interventions [8].

Pharmacological treatment is part of a comprehensive approach, but it is important to classify and evaluate pain before deciding on pharmacological or non-pharmacological therapy to adapt the treatment to the individual [7, 8, 17]. In the management of chronic pain, non-pharmacological interventions have a prominent role, but also pharmacological interventions are recommended if there is a need for that, or combinations thereof [7].

Procedural pain and anxiety can be minimized by informing the children about what is going to happen, as it is important for them to have a sense of control [1, 8]. Active and passive distraction in the form of pictures, music, computer games, controlled deep breathing and guided imagery are techniques used to direct children’s attention away from the procedure [1, 18]. Pharmacological treatment for procedural pain includes local anaesthesia, paracetamol, non-steroidal anti-inflammatory drugs (NSAIDs), opioids, nitrous oxide and sedation [1, 8, 19].

Pain intensity rating is an essential part of pain management for identifying the presence of pain, as well as for indicating and evaluating pain treatment [7, 8, 16]. Self-reporting by patients should be the primary basis for pain assessment [8, 16]. Children aged over 5 years are capable of self-rating their pain intensity [20]. Pain levels should be assessed both at rest and during movement, as the latter can have major effects on a patient’s ability to participate in rehabilitation and return to normal function [8, 21]. To facilitate pain management and communication between healthcare professionals in hospitals, pain and response to treatment should be routinely monitored and clearly documented in the medical records [22].

More knowledge of children’s own pain experience is needed [23]. There is also a gap in the literature regarding children’s self-reported pain both at rest and during movement, and few studies have investigated self-reported pain in hospitalized children. Most of the studies concerning the prevalence of pain amongst children and adolescents are from Canada and the USA with few conducted in Europe.

The aim of this study was to describe the prevalence of pain, self-reported pain intensity at rest and during movement, pain management and compliance with pain treatment guidelines in children and adolescents receiving hospital care. Furthermore, we also wanted to examine the self-reported statements of children and adolescents about what provides pain relief, their pain reports to staff members and how often staff members asked them about pain.

## Methods

### Design and setting

This cross-sectional quantitative study was conducted at a county hospital in western Sweden providing both planned and emergency care. The hospital is responsible for all in-patient care for children living in this area, with a population of 340,000 people. The study was performed for 15 days spread over a 3-month period. Participants were recruited after being identified as having reported any pain during their hospital stay by the nurse responsible for their care. The hospital had pain treatment guidelines for healthcare professionals.

### Participants

The study is based on a convenience sample of children (in this study “children” refers to both children and adolescents) aged 6–18 years who had experienced pain at any time during their hospital stay. They who had been in-patients for at least 24 hours or attended a surgical day care unit were invited to participate. The age of 6–18 years was chosen because that they can accurately self-report their pain, and also that the numerical pain scale is valid for these ages. Exclusion criteria were inability to speak and understand Swedish, cognitive disability, isolation because of infection and critical illness. The evaluation of these factors was done by the paediatric nurse responsible for the patients and was based on medical diagnoses.

Out of 74 eligible children, 69 participated, 53 of whom were in-patients and 16 had undergone an intervention in the surgical day care unit. Two declined participation and three were excluded because of illness on the day of the interview.

### Ethical considerations

The patients were informed about the study orally and in writing. For children aged ≤14 years, their parents’ oral and written informed consent was required, while they themselves provided oral consent. The oral and written information for the younger children was more simply formulated than that for those aged 15–18. Participants aged 15–18 provided oral and written consent themselves. The study was approved by the Regional Ethics Board in Lund (No. 2013/376), Sweden.

### Data collection

Demographic and clinical data, such as age, sex, main diagnosis, other diagnoses that could cause pain, documentation of pain and prescribed analgesic treatment, were obtained from medical records. A structured, verbally administered questionnaire was used to obtain participants’ pain reports. The questions, which were based on those used by Taylor et al. [24], were: How do you rate the highest pain intensity you experienced during the past 24 hours? How do you rate your pain at rest? How do you rate your pain during movement, such as when you are walking around or changing position in bed? The rating was then done on a pain rating scale (see details below). We also asked if they had used a pain rating scale during their hospital stay and if they had experienced procedures as painful. We gave examples such as examination, treatment, and needle procedures. They were also allowed to provide narrative information if they wished.

The children were asked about what they considered alleviated pain. If they had difficulty indicating this, the following alternatives were suggested to them; analgesics, conversation with their parents or staff members, heat/cold therapy, physical activity, music, computer games or “other”. They were also questioned about whether staff members asked them about pain and whether they informed a member of staff when in pain. To collect this information, we gave them the response alternatives; “often”, “sometimes” and “no”.

For the self-rating of pain intensity, we used the 4-point Pain Word Rating Scale (PWRS) comprising “no pain”, “mild pain”, “moderate pain” and “severe pain”. These words can be used and understood by children from 5 to 6 years of age [20]. The PWRS is validated for individual assessment [25] and has been used elsewhere [12, 24, 26]. We also employed a Numerical Rating Scale (NRS), ranging from 0 = “no pain”, 1–3 = “mild pain”, 4–6 = “moderate pain”, and 7–10 = “severe pain”, which is widely accepted for use in ≥8-year-olds [8, 27] and has strong validity and reliability from the age of 6 [28]. The PWRS and NRS were presented on the same side of the instrument and the scale that best suited the child was used. Some 6-year-olds preferred the Coloured Analogue Scale (CAS), a 10-cm line depicted as a colour transition. The CAS has excellent reliability and validity for children aged ≥5 years [20]. The CAS was on the back of the instrument on which the other two scales were presented. The option for the children to choose scale was considered important to get the best circumstances for each child to give a valid report.

Data were collected through a structured interview conducted by two of the researchers. One of the researchers spoke to the children, while the other recorded the answers. Each child’s condition and cognitive capacity determined the speed of both the replies and the rating procedure. The parents were always present in the room beside the child for those aged ≤14 and on most collection occasions also with those aged 15–18, who were asked if they wished to have a parent present. The interview started with a broad open question to the child asking whether she/he was in pain and, if so, about the location and duration of the pain. The child was then instructed how to use the pain rating scale and allowed to choose between the PWRS, NRS or CAS to indicate pain intensity. Two children chose the CAS. The structured, verbally administered questionnaire was used to obtain all the issues about the children’s pain reports.

### Data analysis

The participants were divided into two groups based on their pain condition: acute pain (from surgery or an acute, painful disorder) and chronic pain (lasting > 3 months). A few children with chronic pain had undergone an intervention during their hospital stay, but as chronic pain was their “main pain”, they were categorized as belonging to the chronic pain group. Patient-reported worst pain in the previous 24 hours, pain at rest, and pain during movement were divided into four categories: “no pain” (NRS 0), “mild pain” (NRS 1–3), “moderate pain” (NRS 4–6), and “severe pain” (NRS 7–10). Data were then grouped into patients who experienced mild pain (NRS 1–3), and moderate to severe pain (NRS 4–10) at rest and during movement. Data from the medical records concerning analgesic prescriptions, documented pain and pain rating values were analysed. Analgesic prescriptions were divided into “as needed” and “fixed schedule”.

Information on what the children found pain-relieving was categorized into six groups; analgesics, conversation, active physical therapy, passive physical therapy, cognitive distraction and “nothing helps”. Their statements about how often staff asked them if they were in pain and whether they informed staff about pain as well as the proportion who had been given the opportunity to use a pain rating scale earlier during the hospital stay was likewise analysed. Data from the children’s reports about procedural pain were analysed.

The results are presented with descriptive statistics, including means with standard deviations (SD), and proportions. The Wilcoxon-signed rank test was used for comparisons. Data management and statistical analyses were performed with IBM SPSS Statistics 21 (IBM Corp., Armonk, NY, USA).

## Results

### Demographic data, care categories, pain categories and reason for care

The mean age of the 69 participants was 12.8 years (SD 3.8) and the sample included more girls than boys. Further information about the participants is presented in Table 1.

**Table 1:** Demographic data, care categories, pain categories and reason for care (*n* = 69)

	N	%
Age		
6–13 years	32	46
14–18 years	37	54
Sex		
Boys	29	42
Girls	40	58
Care category		
In-patient care	53	77
Day-surgery	16	23
Pain category		
Acute pain	53	77
Chronic pain	16	23
Reason for care		
Trauma/fracture	11	16
Arthritis/musculoskeletal pain	4	6
Abdominal pain	8	12
Surgery/minor intervention	30	43
Medical care	16	23

### Pain prevalence

**Fig. 1:**
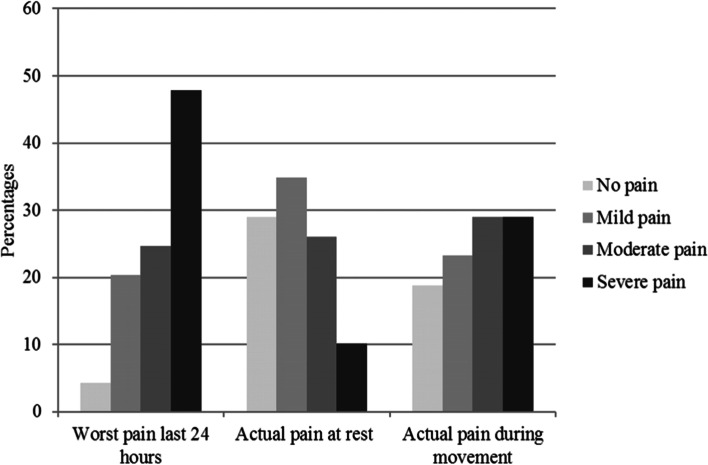
Percentages of children reporting pain severity as measured by the NRS/PWRS or CAS, for worst pain in the past 24 hours and actual pain at rest and during movement

Three children (4%) reported that they had been completely pain-free during the previous 24 hours, while 14 (20%) had experienced mild pain, 17 (25%) moderate pain and 33 (48%) severe pain. Two children did not answer. At the time of the interview, 25 (36%) children reported moderate to severe pain at rest, while the corresponding figure during movement was 40 (58%) (Fig. 1).

Children with acute pain rated their pain significantly (*p* < 0.001) higher during movement than at rest (Table 2). Those with chronic pain reported greater variation of pain scores when moving and the difference compared to at rest was not significant (*p* = 0.083).

**Table 2:** Pain ratings in relation to pain category at time of the interview

	Pain category		
Pain rating	Acute pain *n* = 53 *n* (%)	Chronic pain *n* = 16 *n* (%)	All pain *n* = 69 *n* (%)
**Pain at rest**			
No pain	16 (30)	4 (25)	20 (29)
Mild pain	18 (34)	6 (38)	24 (35)
Moderate pain	15 (28)	3 (19)	18 (26)
Severe pain	4 (8)	3 (19)	7 (10)
**Pain during movement**			
No pain	8 (15)	5 (31)	13 (19)
Mild pain	14 (26)	2 (12)	16 (23)
Moderate pain	18 (34)	2 (12)	20 (29)
Severe pain	13 (25)	7 (44)	20 (29)

### Characteristics of children with severe pain at rest and/or during movement

Twenty of the children (29%) with a range of conditions, experienced severe pain at rest and/or during movement. Of these, seven had undergone surgery, while the others had conditions such as: chest pain, abdominal pain, arthritis, mononucleosis, musceloskeletal pain, head injury, trauma, or fractures. Seven of the children (10%) experienced severe pain both at rest and during movement; three due to abdominal pain, one with mononucleosis, one with head injury and two because of orthopaedic surgery. All twenty children rated their pain score higher during movement than at rest, and all had analgesic prescriptions as needed, or as scheduled, or both (12 children). Twelve children mentioned that various non-pharmacological methods were used in addition to analgesics. Six of those with severe pain had used a pain rating scale during their hospital stay.

### Procedural pain

Thirty children (43%) reported that they had experienced procedural pain in addition to their main pain classification. Needle procedures such as blood draw (10 children) and insertion of a peripheral intravenous catheter (14 children) were the most frequently reported sources of procedural pain. Other sources were lumbar puncture, abdominal palpation, and examination. Fifteen children stated that topical anaesthesia of the skin prior to needle procedures provided relief. A small number reported painful repeated attempts at needle procedures, including one lumbar puncture.

### Children’s own statements about pain relief

Fifty-one children (74%) reported that analgesics provided pain relief. Forty (58%) stated that different non-pharmacological methods were helpful, such as supportive conversations with parents and healthcare professionals (26%), active physical activity (dancing, physiotherapy, movement, and massage) (9%), and passive physical activity (heat/cold, pressure on the painful area, immobilization, and rest) (20%). Regarding cognitive distraction (33%), some answered that they tried to think of other things, while the coping strategies of others included taking part in play therapy on the ward, creating with their hands, using mobile phones, listening to music and watching television. Five children had difficulty achieving relief and stated that nothing alleviated their pain.

### Children’s pain reports to staff and frequency of staff asking them about pain

Of the 69 participants, 49 (71%) reported that they told staff when they were in pain, eight (12%) stated that they sometimes told staff, six (9%) that they did not tell staff, while five (7%) said they preferred telling their parent about their pain. One did not answer.

Forty-three children (62%) indicated that staff frequently asked them if they were in pain, while six (9%) said that staff sometimes asked, and 19 (28%) that staff rarely asked. One child did not answer.

### Documentation of pain and pain rating

Documentation in the medical records of pain at some time during the past 72 hours was retrieved for 43 children (62%). Nurses had documented pain rating values in the medical records of 27 children (39%). Nineteen children (28%) stated that they had already used a pain rating scale during their present hospital stay.

### Analgesic prescription

The children were prescribed analgesics at fixed intervals and/or as needed (Table 3). Multimodal analgesics, including two or more medications on a regular basis, were prescribed for all 10 children who received opioids at fixed intervals, combined with paracetamol and/or NSAIDs. Another nine children were prescribed paracetamol and NSAIDs on a regular basis. Therefore, 19 (28%) children were on regular multimodal medication.

**Table 3:** Prescription of analgesics on fixed schedule or as needed and type of pain

	Acute pain	Chronic pain	Acute and chronic pain
	*n* = 53	*n* = 16	*n* = 69
	*n* (%)	*n* (%)	*n* (%)
Paracetamol			
Fixed schedule	29 (55)	6 (38)	35 (51)
As needed	18 (34)	2 (12)	20 (29)
NSAIDs			
Fixed schedule	15 (28)	3 (19)	18 (26)
As needed	11 (21)	1 (6)	12 (17)
Opioids			
Fixed schedule	5 (9)	5 (31)	10 (14)
As needed	19 (36)	6 (38)	25 (36)

## Discussion

In this descriptive study of hospitalized children, a high proportion who had already stated that they had pain, reported moderate to severe pain at rest or during movement. This may indicate that many children were undertreated. The study reveals a gap between pain management in hospitalized children and evidence-based knowledge [7, 8], highlighting a need for improvement. Adequate pain management is of the utmost importance, as acute pain can lead to consequences such as chronic pain [2, 3] and post-traumatic stress symptoms (PTSS) [4].

Other studies have reported similar findings with moderate to severe pain intensity during the previous 24 hours in 24–50% of hospitalized children [9–11, 13–15]. These studies were performed in different age groups, from infants to adolescents. Our study included children aged 6–18 years as we wanted to collect information from the patients themselves. It has previously been demonstrated that children’s pain is underestimated by parents and healthcare professionals [26, 29, 30]. In our study, pain during movement was higher (58%), than pain at rest (36%). It is of great importance to also assess pain during movement, as relatively well controlled pain at rest can become severe during movement, or when doing specific activities [8]. Other studies examining pain in hospitalized children have not presented specific data on pain intensity during movement, which data are of importance for better understanding children’s experiences of pain.

Children in our study reported needle procedures as painful, but also stated that local anaesthesia provided relief. Procedural pain is a significant problem for hospitalized children as documented in previous studies [10, 15, 26, 31]. A few children in our study reported that repeated painful attempts had been made at needle procedures. One of them had undergone a lumbar puncture and her/his wish to discontinue the intervention had not been respected. Such episodes can frighten a child and weaken her/his trust in healthcare professionals and treatment [1] or cause PTSS [4, 32]. It is of the utmost importance for healthcare professionals to adopt an ethical approach to younger patients and respect their integrity. A study by Bray et al. [33] suggests that a balanced approach is facilitated by a clinical pause, which can give healthcare professionals the time to consider children’s expressed wishes and find alternatives. In accordance with the UN Convention on the Rights of the Child, a key principle is that all children have the right to express their opinion, be heard and respected [34].

In the present study, the majority of participants reported that analgesics alleviated their pain. This is in line with other studies reporting that analgesics provided relief in > 50% of children [9, 24, 26, 29]. The children in our study also stated that different non-pharmacological methods relieved the pain, as shown in other studies where children and parents appreciated initiatives other than medication for providing relief [9, 10, 15, 26, 31, 35]. It is important for staff to be aware of the value of these methods. Five children in our study stated that nothing had helped, indicating the need to focus on individual pain management. A major aim of pain treatment is to eliminate pain-associated suffering, which occurs when the pain is overwhelming and leads to the patient experiencing a loss of control [22]. Paediatric patients have expressed that they want to get interventions (pharmacological or non-pharmacological) to alleviate pain [15].

The fact that 20 children in our study reported severe pain (NRS 7–10) at rest or during movement and that seven of them had a high pain-score both at rest and during movement, despite all of them having had a prescription for analgesics, indicates that the treatment response was not followed up. The presence of severe pain should have been met by further pharmacological and/or non-pharmacological interventions from healthcare professionals [7, 8].

The finding that six children from our population did not tell staff about their pain and that five told their parents instead, highlights the importance of healthcare staff regularly asking children about pain. Paediatric patients who are stoic or depressed may not report or show expected pain behaviour, which can be misinterpreted if healthcare professionals do not specifically ask about pain [22]. One review article reports that teenagers sometimes try to act like adults and therefore do not want to complain [36]. This could also explain why some of our participants did not tell staff about their pain.

More than half of the children reported that staff often asked them if they were in pain. However, if pain assessment had been used more regularly in combination with questions about pain, the chances of identifying those with severe pain would have increased. Twenty-eight percent of children stated that staff rarely asked them about pain. This agrees with the findings of another study, where 24% said that nurses did not talk to them about pain relief as often as they would have liked [37].

Our study shows that only 28% of the children had used a pain rating scale during their hospital stay. Furthermore, documentation of pain and pain rating values in the medical records was insufficient. Other studies have likewise revealed a lack of documentation about pain and pain assessment [10, 15, 24, 29, 38]. It is reasonable to believe that fewer children in our study would have suffered moderate to severe pain if pain assessment had been carried out more systematically. In order to adequately treat pain, ongoing assessment of its presence, severity and treatment response is essential [22].

About one-third of patients in our study were on a regular multimodal analgesic regimen. The findings indicate that a greater proportion should have received two or more drugs regularly, as a substantial number of children had moderate to severe pain at rest or during movement. A regular multimodal analgesic regimen in accordance with guidelines [7, 8] would probably have contributed to lower “worst pain” levels in these patients.

Fixed schedule paracetamol was prescribed in half of the children in our study. This proportion is larger than the 19–45% reported elsewhere [10, 11, 13, 15]. Twenty-six percent of our participants received fixed schedule NSAIDs. Administration of NSAIDs is associated with a risk of side effects, such as gastrointestinal bleeding, anaphylactic reaction and decreased renal function, but these are rarely seen in children [39]. It is likely that physicians are reluctant to prescribe NSAIDs to children, although their analgesic effect is almost comparable to that of strong opioids [40].

Of our participants, 36% received opioids “as needed” and 14% as a fixed-schedule medication. The level of prescription of opioids in other studies is both lower [9, 10, 15] and higher [13] than our findings. More children in our study would have benefited from a fixed schedule of opioids, because “as needed” drugs are frequently administered sporadically [13]. As our findings indicate, children do not always tell when they are in pain, and some have difficulty communicating. Other studies have also revealed that analgesic prescriptions for hospitalized children are inadequate [15, 31].

A study from Canada reported on an improvement programme that led both to a lower proportion of children experiencing severe pain and to increased documentation of pain [41]. Another study demonstrated higher standards and improved pain management after enlisting an acute pain team [42]. There were no such teams in the hospital in our study. Thus, the results might have been better if experts had been available to provide specialist pain advice. Children are vulnerable and have difficulty communicating their pain, which means that they require extra attention [21, 43].

A strength of this study is that the data are based on patient self-reports. Another strength is that the children were asked about their pain both at rest and during movement, thus providing more comprehensive data. The present study captured several aspects of children’s own perspective on their pain experience during a hospital stay. The number of participants is a limitation, especially with regard to the possibility to do analyses on associations with background factors. However, on the days on which the data collection took place the majority of in-patients were aged under 6 years and thereby too young for this study. There is also a limitation with the exclusion of patients with critical illness and infectious diseases, and the generalizability should be seen with this in mind.

## Conclusions

Despite the availability of evidence-based guidelines, pain is still undertreated in children. Furthermore, pain assessment and documentation are deficient, and drug prescription does not adequately comply with guidelines. Pain levels should be assessed both at rest and during movement to obtain a more comprehensive picture of the pain experienced. Response to treatment should be monitored routinely to prevent undertreatment of pain. Professional communication and an ethical stance are of vital importance for pain management in children and adolescents receiving hospital care. Different non-pharmacological methods are valuable for children with pain in addition to pharmacological interventions. This study shows that it is important to evaluate and improve pain care also outside specialised tertiary clinics. Future research could focus on finding factors that reduce the gap between guidelines and the clinical practice in pain treatment.

## Data Availability

All data generated or analysed during this study are included in this published article.
